# Rapid and effective malaria control in Cambodia through mass administration of artemisinin-piperaquine

**DOI:** 10.1186/1475-2875-9-57

**Published:** 2010-02-23

**Authors:** Jianping Song, Duong Socheat, Bo Tan, Prak Dara, Changsheng Deng, Sreng Sokunthea, Suon Seila, Fengzhen Ou, Huaxiang Jian, Guoqiao Li

**Affiliations:** 1 Research Centre for Qinghao, Guangzhou University of Chinese Medicine, Guangzhou, PR China; 2 National Centre for Parasitology, Entomology and Malaria Control, Phnom Penh, Cambodia; 3 Department of Health, Kampong Speu, Cambodia

## Abstract

**Background:**

Previous efforts to eradicate malaria parasites, particularly *Plasmodium falciparum*, have failed in part due to the emergence of drug resistant parasites and mosquitoes resistant to insecticides. Using an artemisinin-based combination therapy (ACT) that kills parasites quickly, a strategy was designed to eliminate the source of transmission by mass treatment of human populations in malaria-endemic areas Cambodia.

**Methods:**

A combination drug of artemisinin and piperaquine given with low doses of primaquine was used to eliminate all stages of parasites from human carriers.

**Results:**

In a pilot study, mass administration of artemisinin-piperaquine (two tablets of 62.5 mg artemisinin and 375 mg piperaquine for adults aged ≥16 years at 0 and 24 hrs; 1.5 tablet for children aged 11-15 years; and one tablet for children aged 6-10 years) and primaquine (9 mg for adults, at 10 day intervals for 6 months) was carried out in 17 villages (3,653 individuals). Parasite rates were dramatically reduced from 52.3% to 2.6% after three years. The *P. falciparum *rate in children decreased from 37.0% to 1.4%, reaching 0% in eight of 17 villages. In a second field study, that included one additional mass treatment of artemisinin-piperaquine, the *P. falciparum *rate in children was reduced from 20.8% to 0% within six months. No major adverse effects were observed.

**Conclusions:**

Mass administration of artemisinin-piperaquine and low doses of primaquine can be an effective, safe, and affordable strategy for efficiently eliminating malaria parasites in human carriers and interrupting parasite transmission. This study provides important information for future strategies for the eradication of malaria.

## Background

Malaria has been eliminated from some formerly endemic regions of the world, mainly in more temperate zones including countries in Europe, North America, some of the former Soviet Republics, and some island nations. Improvement in public health, efforts in treating malaria patients, and mosquito control measures were some key factors for the success of malaria elimination. Unfortunately, malaria control programmes have been less successful in many developing countries in the tropics and subtropics. Lack of resources for disease management and the emergence of drug resistant parasites and insecticide resistant mosquitoes contributed to the failure of many malaria eradication programmes during the era of the Global Malaria Eradication Programme initiated by the World Health Organization in 1955 [1].

Similarly, the goals of the1998 Action Plan to Roll Back Malaria have not been fulfilled in many countries [2]. In China, integrated malaria control programmes, such as mosquito and transmission control, have been in place to eliminate the disease since the late 1950s [3]. However, it took over 30 years to control *Plasmodium vivax *malaria in endemic areas along the Yangtze River [4]. Although *Plasmodium falciparum *malaria has been eliminated in many endemic regions in China, the parasite is still present in Hainan and Yunnan provinces in Southern China after more than 50 years of disease control efforts [5,6].

To reduce or totally eliminate malaria parasite infections from a population, interruption of parasite transmission is critical. One strategy for achieving the goal is to interrupt parasite transmission through mosquito vector control such as insecticide spraying and large-scale distribution of insecticide-impregnated bed nets [7]. Although some successes have been achieved through these efforts, vector control in most endemic regions has had limited success at sustained interruption of transmission, especially when applied in isolation without aggressive drug treatment and prevention strategies. A second approach is to eliminate parasites from the human reservoir with anti-malarial drugs. Unfortunately, many anti-malarial drugs cannot kill sexual stages in a human host and, therefore, cannot block parasite transmission even when blood stage parasites are eliminated and the patient is successfully treated for malaria symptoms. Additionally, the emergence of parasites resistant to anti-malarial drugs has contributed to resurgence of malaria cases in recent decades [8].

Rapid-acting artemisinin combination therapy (ACT) provides a potential tool for malaria control and eradication by removing the source of malaria transmission through eliminating parasites in the human populations. This strategy has been shown to be effective previously, particularly when combined with anti-vector methods [9-11]. This approach is also consistent with previous experiences in eliminating malaria parasites from places with endemic malaria or with malaria outbreaks (personal communication with Qilin Huang, Institute of Anti-Parasitic Diseases, Guangdong Province, China). Where malaria rates are low, malaria outbreaks have occurred because of transmission from undiagnosed and untreated imported carriers. Indeed, the successful eradication of malaria from regions such as North America was achieved largely through eliminating malaria parasites from human populations, not by eliminating their mosquito vectors.

To investigate the feasibility of eradicating malaria parasites from endemic regions through eliminating the sources of host transmission, two pilot studies were conducted in Cambodia during December 2003 to December 2006. Here results are presented from the studies testing a hypothesis that mass treatment with an ACT followed by primaquine to block transmission is an effective method for reducing and/or arresting malaria transmission in endemic area.

## Methods

### Study population

**Figure 1:**
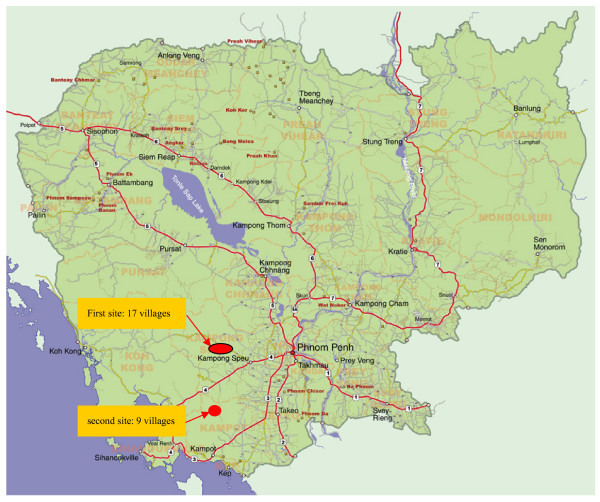
**Study sites in Cambodia**. First site, 17 villages in Kampong Speu province; Second site, 9 villages in Kampot province.

The population for the first study consisted of 3,653 individuals from 17 villages in Kampong Speu province, Cambodia (Figure 1). The second study population included 2,387 individuals in nine villages in Kampot province (Figure 1). The majority of the study populations were of Khmer ethnic origin. The studies were approved by Institutional Review Boards of the Ministry of Health, Cambodia and Guangzhou University of Chinese Medicine, Guangzhou, People's Republic of China.

### Drug administration

Village Malaria Volunteers (VMV) were recruited to distribute drugs and monitor drug administration. Anti-malarial drugs were given to every individual simultaneously in the villages. A complete two-dose therapy of artemisinin-piperaquine was given to the whole population without obtaining individual infection information. Adults (≥16) were given two tablets of a co-formulated artemisinin and piperaquine (artemisinin 62.5 mg and piperaquine 375 mg/tablet, Artequick^®^, Guangzhou, PRC) at 0 and 24 hours. For children aged 6-10 years, one tablet was given at each dose; and for children aged 11-15 years, one and a half tablets were used. For children under 6 years, a complete two-dose artemisinin-piperaquine Granules (artemisinin 24 mg and piperaquine 144 mg/sachet) was administered: two sachets each dose for children aged 5-6 years; 1.5 sachets for children aged 3-4 years; and one sachet for children aged 1-2 years. At the same time, low-dose primaquine tablet (9 mg) for adults was given at 10-day intervals for six consecutive months without obtaining individual G6PD information, to prevent transmission to mosquitoes from infected subjects. The first dose of primaquine was taken with artemisinin-piperaquine. The doses of primaquine for children were: 3/4 tablet for children aged 11-15 years; 1/2 tablet for children aged 7-10 years; 1/3 for children aged 3-6 years; and 1/4 for children aged 1-2 years.

Because some villages still had low parasite rates after the first pilot study, a second mass treatment of artemisinin-piperaquine was added in the second study in Kampot. It was observed that it was critical to have well-trained VMV who could properly administer the drugs. Some of the VMV were replaced to improve drug distribution. In the second study in Kampot, the same procedure was implemented as in the first one, with one additional mass treatment of artemisinin-piperaquine 42 days after the first treatment for villages where the parasite rates remained ≥ 10%. For villages with a positive rate <10%, mass treatment was given only once as in the first study.

All febrile patients were treated at no cost with complete two-dose regimens of artemisinin-piperaquine and one dose of primaquine during the project. No specific vector control measures by other research or charitable organization were identified in the study areas.

### Monitoring parasitaemia

Parasite rates in the whole population and in children (aged < 16 years) were the main indices used to monitor malaria prevalence and treatment effectiveness for the studies.

After the mass treatment programme was started, periodic surveys monitoring parasite positive rates in villages with initial parasite rates of ≥ 20% were carried out every 6 months. Parasite rates in 50 adults and 50 children were determined randomly in every village. In a village with a population less than 100, the number of adults and children checked was not less than 70% of the population. When the population parasite rate in a village was decreased to zero, all negative blood smears of that village were rechecked by another senior microscopist, appointed by both Chinese and Cambodian investigators, to confirm the results. One skilled microscopist was responsible for microscopic examination of blood smears throughout the study so that errors due to variation of diagnostic skill would be minimized.

Children parasite positive rates in three of the nine villages in the second study were surveyed by malaria microscopy on Days 7, 14, 28, 35, 42, 60, 90, 120, 150, and 180.

### Monitoring severe adverse events

Severe adverse events are passively reported by the VMV. No active monitoring takes place.

### Statistical methods

The chi-square test (SPSS 10.0) was used for comparisons of parasite rates with two-tailed significance tested at *P *< 0.05.

## Results

### Survey of initial malaria infection

Human populations were surveyed in 27 villages in Kampong Speu province for malaria parasites before the programme was started. Three different malaria species, *P. falciparum*, *P. vivax*, and *Plasmodium malariae*, were found in the villages.

**Figure 2:**
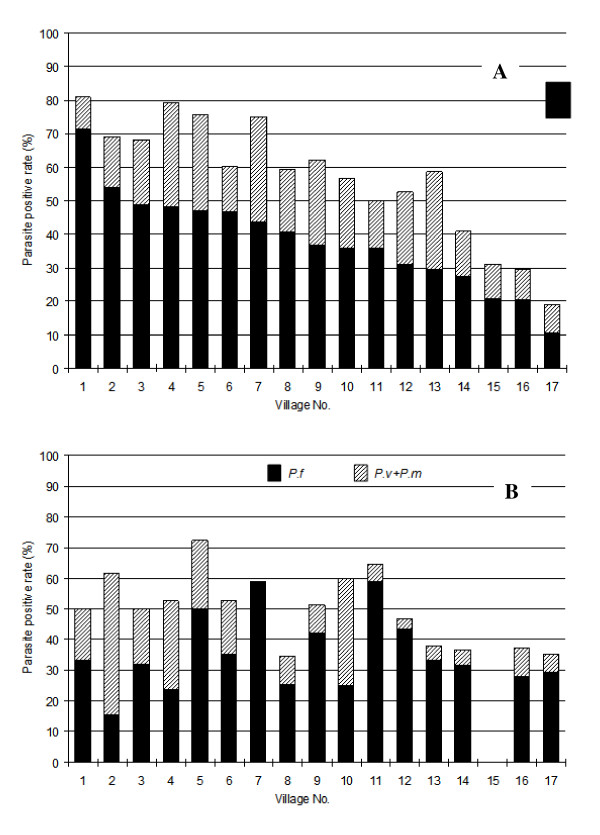
**Parasite rates in children (A) and adults (B) from 17 villages before treatment**. Parasite rates in adults of Village 15 were not obtained.

Among the 27 villages, 17 villages had parasite rates ≥ 20%, six villages had parasite rates 10-19%, and four villages had parasite rates 6-9%. The 17 villages with high parasite rates had 3,653 residents, with parasite rates among children ranging form 19.1% to 81.0% (Figure 2A). In adults, the parasite rates were similar to those of children (p = 0.10, paired *t*-test) (Figure 2B). Among the three species, *P. falciparum *was the most prevalent (68.7% and 58.1%, in the 17 villages with high parasite rates and in the 10 villages with low parasite rates, respectively.); *P. vivax *was the second most prevalent (23.8% and 38.7%) followed by *P. malariae *(7.5% and 3.2%). Interestingly, there appeared to be no correlation between parasite rates in adults and children among the villages. That is, villages having the highest rates in children were not necessarily those with the highest parasite rates in adults. Similarly, the ratios of *P. falciparum *over *P. vivax *and *P. malariae *in children were often quite different from those in adults in each village.

### Survey of glucose-6-phosphate dehydrogenase (G6PD) deficiency rate

A total of 252 villagers (only one from every family) were screened for G6PD deficiency using G-6-PDH Kit Visual Colour, Trinity biotech. The G6PD deficiency rate is 17.1% (43/252); male 18.6% and female 13.3%.

### Dramatic reduction in parasite rates after mass treatment

**Figure 3:**
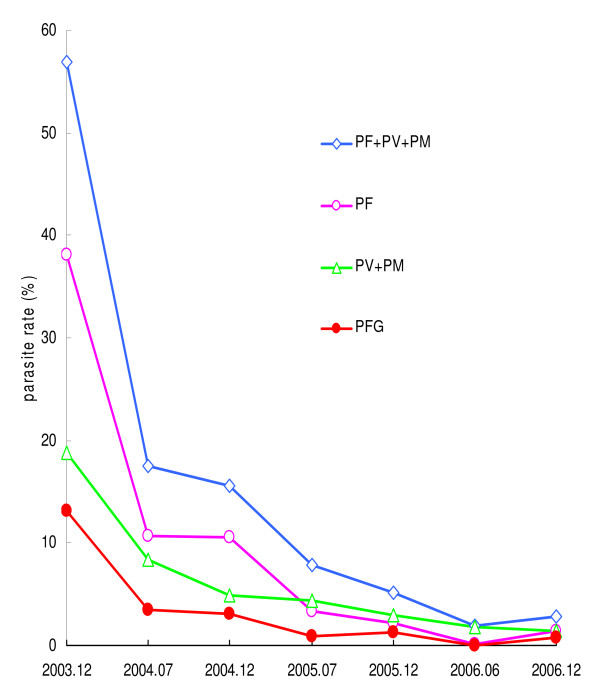
**Reduction in parasite rates in children in the 17 villages**. *P. f, P. falciparum; P. v, P. vivax; P. m, P. malariae; *PFG, *P. falciparum *gametocyte

**Figure 4:**
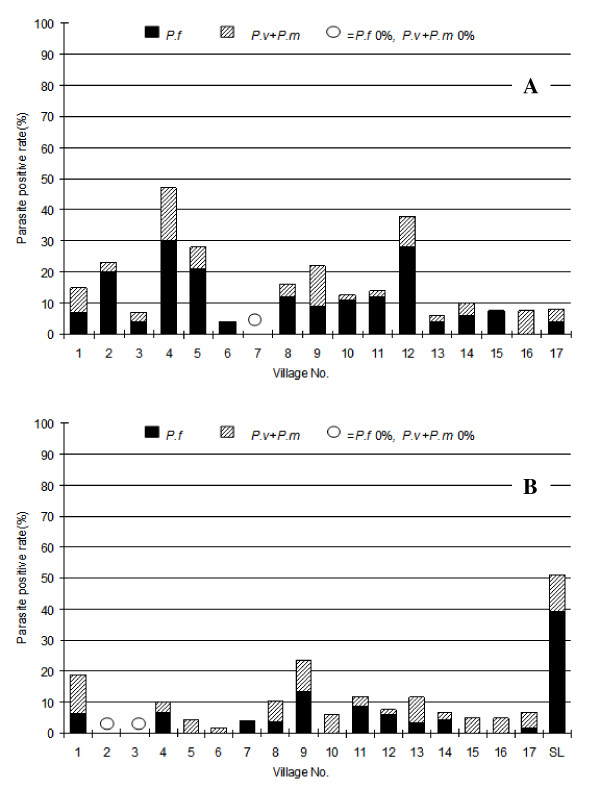
**Parasite rates in children in the 17 villages 12 (A) and 18 (B) months following start of mass drug administration**. Village SL is 3 km from Village 9. The high parasite rate might influence the treatment outcomes of nearby Village 9.

Dramatic reductions in parasite rates were observed in the 17 villages after three years of treatment and monitoring (Figures 3 and 4). Total average parasite rates in the whole population decreased from 52.3% at the beginning of the study to 2.6% at the end of the study (Table 1). In children, the parasite rate decreased from 55.8% to 15.7% (71.9% reduction) in the first year, to 5.3% (90.5%) in the second and to 2.8% (95.0%) in the third year. The *P. falciparum *rate in children decreased from 37.0% to 10.8% (a reduction of 70.8%) in the first year, to 2.3% (93.8% reduction) in second years and to 1.4% (96.2% reduction) by the third year. The combined *P. vivax *and *P. malariae *rate decreased from 18.8% to 4.9% (73.9% reduction) in the first year and to 3.0% (84.0% reduction) in the second year, and 1.4% (92.6% reduction) in the third year (Table 1).

**Table 1:** Average population parasite carriage rates in the 17 villages

Age group	Parasite rates (%)						
	
	2003.12	2004.07	2004.12	2005.07	2005.12	2006.6	2006.12
Children	55.8	17.5*	15.7*	7.8*	5.3*	1.9*	2.8*
	(*N *= 679)	(*N *= 777)	(*N *= 813)	(*N *= 877)	(*N *= 704)	(*N *= 791)	(*N *= 844)

Adults	46.5	NA	10.7*	4.9*	6.3*	NA	2.1*
	(*N *= 415)		(*N *= 796)	(*N *= 616)	(*N *= 366)		(*N *= 436)
Whole population	52.3	-	13.2*	6.6*	5.6*	-	2.6*
	(*N *= 1094)		(*N *= 1609)	(*N *= 1493)	(*N *= 1070)		(*N *= 1280)

* *P *< 0.01 vs. the data obtained in Dec.2003 (before conducting mass treatment); NA, not done.

Reductions in parasite rates for adults were similar to those for children (Table 1). Parasite rates changed from 46.5% to 10.7% (77.0% reduction) in the first year, to 6.3% (86.5% reduction) in the second year, to 2.1% (95.5% reduction) in the third year. The *P. falciparum *rate decreased from 34.2% to 1.6% (95.3% reduction) after three years; the combined *P. vivax *and *P. malariae *rate decreased from 12.3% to 0.5% (95.9%). It should be noted that the *P. falciparum *positive rate in children in eight of 17 villages decreased from an average of 46.5% to 0% 18 months after the start of the mass administration programme.

### Exceptions in rate reductions

Although the mass treatment worked well in most of the villages, not all villages had similar reductions in parasite rates after one year (Figure 4). Village 2, 4, 5, 9, and 12 still had parasite rates higher than 20% 12 months after the baseline measurements (Figure 4A and Table 2). Interviews with villagers indicated that the drugs might not be distributed properly through VMV. Consequently, some VMV were replaced and a second treatment with artemisinin-piperaquine was given to residents in the four villages, which resulted in significant reduction in parasite positive rates, with three out of four (village 2, 4 and 12) having 0% of parasite rate 1.5 years after second treatment (Table 3).

**Table 2:** Parasite carriage rates of *P. f *and *P. v + P. m *in the17 villages

Age group	*P. falciparum *rates (%)							*P. vivax *+ *P. malariae *rates (%)						
		
	2003.12	2004.07	2004.12	2005.07	2005.12	2006.6	2006.12	2003.12	2004.07	2004.12	2005.07	2005.12	2006.6	2006.12
Children	37.0	9.1*	10.8*	3.4*	2.3*	0.13*	1.4*	18.8	8.4*	4.9*	4.4*	3.0*	1.8*	1.4*
	(*N *= 679)	(*N *= 777)	(*N *= 813)	(*N *= 877)	(*N *= 704)	(*N *= 791)	(*N *= 844)	(*N *= 679)	(*N *= 777)	(*N *= 813)	(*N *= 877)	(*N *= 704)	(*N *= 791)	(*N *= 844)

Adults	34.2	NA	4.9*	1.6*	2.5*		1.6*	12.3	NA	5.2*	3.1*	3.8*		0.5*
	(*N *= 415)	NA	(*N *= 796)	(*N *= 616)	(*N *= 366)		(*N *= 436)	(*N *= 415)		(*N *= 796)	(*N *= 616)	(*N *= 366)		(*N *= 436)

Whole Population	35.9		8.2*	2.7*	2.3*		1.5*	16.4		5.0*	3.9*	3.3*		1.1*
	(*N *= 1094)	-	(*N *= 1609)	(*N *1493)	(*N *= 1070)		(*N *= 1280)	(*N *= 1094)	-	(*N *= 1609)	(*N *= 1493)	(*N *= 1070)	-	(*N *= 1280)

* P < 0.01 vs. the data obtained in Dec.2003 (before conducting mass treatment)

**Table 3:** Changes in children *P. falciparum *carriage rates in village No.2, No.4, No.5 and No.12

**Village No**.	*P. falciparum *carriage rate of children (%)				
	
	2003.12	2004.07	2004.12	2005.07	2005.12
02	53.8	20.0	20.9	0*	0

04	48.3	40.9	29.7	6.7*	0

05	47.2	12.2	21.6	0*	1.9

12	30.9	11.1	26.3	6.0*	0

* *P *< 0.01 vs the data obtained in Dec.2003 (before conducting mass treatment)

The second mass treatment with artemisinin-piperaquine was not implemented in village 9 because of logistical problems. The decrease in the parasite rate in children was 64.2% (compared to ~93% in other villages) 1.5 years after treatment. This could be mainly due to the interference from a nearby village (SL), a highly endemic village in a neighbouring province that was not included in the programme. The village was only three kilometres away from village 9 and the parasite rate in children in the nearby village was 51.2%, 77.3% of which was *P. falciparum *(Figure 3B). Therefore, the SL village could be considered as a control village to compare the parasite positive rate over the same period.

### Reduction in gametocyte rate

*Plasmodium falciparum *gametocyte rates dropped significantly (Figure 3), particularly among children (reduction from 13.1% to 0.8% after 3 years, Table 4).

**Table 4:** Changes in *P. falciparum *gametocyte carriage rates in the 17 villages

Age group	*P. falciparum *gametocyte carriage rates (%)				
	
	2003.12	2004.12	2005.12	2006.6	2006.12
Children	13.1	3.1*	1.2*	0	0.8
	(*N *= 679)	(*N *= 813)	(*N *= 704)	(N = 791)	(*N *= 844)

Adults	10.2	1.4*	1.3*	-	0.7
	(*N *= 415)	(*N *= 796)	(*N *= 796)		(*N *= 436)

Entire population	12.0	2.3*	1.2*	-	0.8
	(*N *= 1094)	(*N *= 1609)	(*N *= 1070)		(*N *= 1280)

* *P *< 0.01 vs the data obtained in Dec.2003 (before conducting mass treatment)

### Improved parasite rate reduction with a second mass treatment

**Figure 5:**
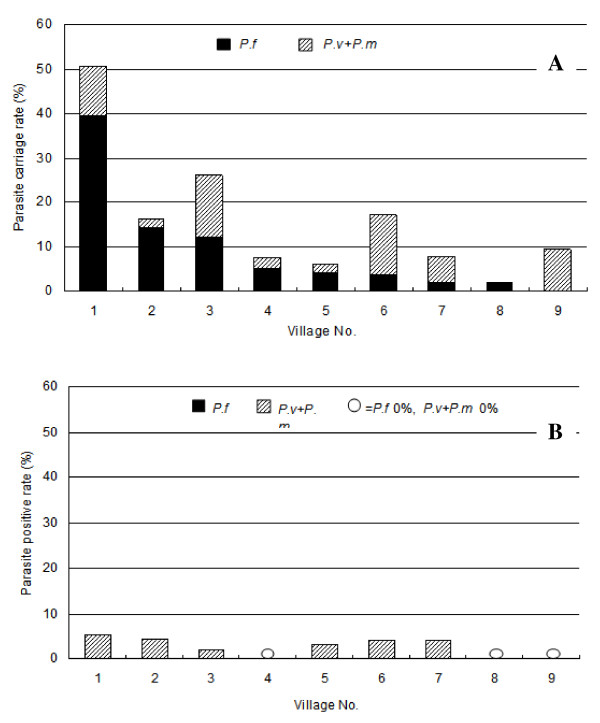
**Parasite rates in children in the 9 villages before (A) and 6 months after mass treatment (B)**.

**Figure 6:**
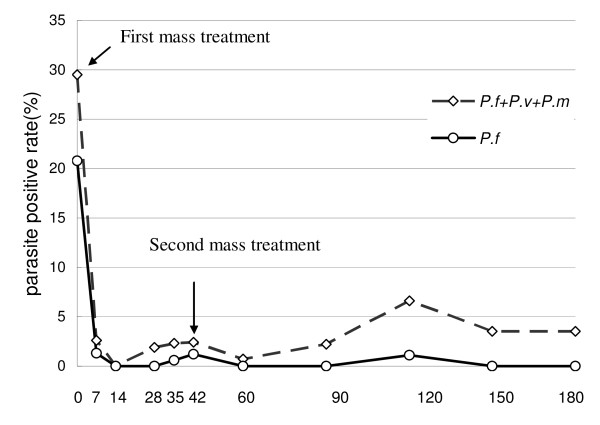
**Changes in parasite rates in children in the 3 villages in the second study with two mass drug administrations**.

To achieve parasite clearance from the population in a shorter time and reduce the risk that parasite carriers could spread malaria infection, and to eliminate parasites from people who experienced recrudescence after a single treatment, we conducted a second study with one additional mass treatment. Nine villages in Kampot region (Figure 1) with parasite rates ranging from 2.0% to over 50.5% (Figure 5A) were selected. Six months after initiation of the second round of mass treatment, parasite rates decreased dramatically (Figure 5B). Significantly, three of the nine villages had 0% *P. falciparum *positive rates at Days 60, 90, 150, and 180 (Figure 6). While adverse events were not monitored actively, no untoward drug reactions were passively reported by VMVs during the projects.

## Discussion

This study demonstrated that mass administration of artemisinin-piperaquine plus primaquine was a practical and effective way to quickly reduce malaria parasite infection in general human populations. The method involves rapidly eliminating the malaria parasites from malaria cases and asymptomatic carriers, including both sexual and asexual stages. This was achieved by mass treatment of human populations with a rapid-acting ACT to eliminate against asexual stages and primaquine to kill sexual and liver stages. Three years after starting the mass administration scheme, the *P. falciparum *rate in children in the 17 villages dropped by 96.2%. Moreover, eight of the 17 villages had parasite rate decreased from 46.5% on average to 0%. The *P. falciparum *rates in four of these eight villages stayed at 0% based on surveys at 18, 24, and 30 months.

The success of the programme depended on well-trained local health workers and village volunteers. It was also important to enlist the cooperation of village leaders and to educate the general population to encourage active participation for the mass treatment to be successful.

This study supports the idea of administering a second mass treatment twice at 42 days because it is difficult to enrol every person in the villages in the first mass treatment, and to eliminate recrudescent infections. An *Anopheles *mosquito can live up to 3-4 weeks or even longer in ideal situations [12]; and they can bite and re-infect previously treated individuals if only one mass treatment is given. In addition, mass treatment in the dry season when the mosquito population is lowest should be considered. These measures were in fact carried out in the second study in which the P. *falciparum *positive rate in children dropped from 20.8% to 0-1.2% within the first 1-5 months and to 0% at the 6th month.

Malaria control by attacking the mosquito vector and by using bed nets, even if insecticide-impregnated, have had limited success worldwide over the past 50 years. So there was a growing realization that effective control of malaria needs new strategies, including the availability of better drugs and a detailed understanding of the epidemiology of local malaria in endemic area [13]. Mass drug administration through a state-wide quinine distribution has been used to control malaria in Italy in 1900s [8] with success in reduction in malaria mortality but failure to significantly reduce transmission. Mass drug administration schemes should ideally use more than one drug, preferably combinations including a rapid-acting schizonticidal drug, such as an artemisinin, that can also kill parasite quickly to reduce the number of gametocytes in the blood, as well as a drug such as primaquine that can kill sexual and liver stages to prevent transmission [11]. The effect of artemisinin on malaria transmissibility relies on its ability to reduce markedly the formation of mature, infective gametocytes [14]. The use of multiple drugs can also reduce the chances of selecting drug resistant parasites.

The studies showed that malaria control through elimination asexual and sexual parasites in carriers (malaria patients and asymptomatic persons) through mass administration of artemisinin, piperaquine, and primaquine was effective. Safety monitoring was not performed systematically as would be done in a controlled clinical trial, but there was no indication of clinically significant adverse drug reactions. Higher doses of primaquine can cause haemolytic anaemia in individuals with G6PD deficiency, but the lower doses used in this programme are thought to be safe, based on extensive experience in malaria elimination campaigns in past decades. The cost of this approach is also acceptable since the prices of artemisinin-piperaquine and primaquine are relative low ($2/treatment/person) and payments for local VMVs is affordable.

Although this three-year mass drug administration project had dramatically reduced parasite rates to very low levels, including some villages with no detectable parasites, residual parasites may act as parasite source of future outbreaks. Another round of treatment is believed to eliminate the residual parasites from the villages. Another potential issue is that parasite carriers in nearby villages may reintroduce the parasites into villages that were clear of parasites. This concern can be resolved if a national wide mass treatment campaign is conducted. A third concern is that mass treatment may lead to the emergence of drug resistant parasites. The mass drug administration programme used three different drugs at the same time, which should greatly reduce the chance of encountering drug resistant parasites.

## Conclusions

Mass drug administration with artemisinin-piperaquine and primaquine can be considered as an alternative strategy for malaria control, and, in combination with other anti-malarial measures, may provide a tool for malaria elimination and eradication.

## Competing interests

The authors declare that they have no competing interests.

## Authors' contributions

JS organized the programmes and draft the manuscript; DS participated in and supervised the programmes; BT participated in the projects and analyzed the data. PD participated in the Kampong Spur project; CD did the field work and analyzed the data; PS participated in Kampot project; S Sokunthea, FO, HJ checked the blood smear; S Seila did the field work; and GL conceived the study, and participated in its design and coordination and helped to draft the manuscript. All authors read and approved the final manuscript.
